# The clinical effectiveness and safety of Zone III REBOA for resection of sacropelvic tumors in patients older than 70 years

**DOI:** 10.1186/s12957-024-03398-2

**Published:** 2024-05-03

**Authors:** Zhiqing Zhao, Jichuan Wang, Jianfang Niu, Sen Dong, Jingtian Shi, Taiqiang Yan, Wei Guo, Rongli Yang, Xiaodong Tang

**Affiliations:** 1 Musculoskeletal Tumor Center, Peking University People’s Hospital, Beijing, China; 2 Department of Orthopedic Surgery, Peking University First Hospital, Beijing, China

**Keywords:** Aortic occlusion, Hemodynamic, Sacropelvic tumor, Hemostasis

## Abstract

**Background:**

REBOA is a method used to manage bleeding during surgery involving sacropelvic tumors. Nevertheless, studies on the use of REBOA among elderly people are lacking. The aim of this research was to investigate the efficacy and safety of Zone III REBOA in patients aged more than 70 years.

**Methods:**

A comparative study was conducted using case-control methods. A group of patients, referred to as Group A, who were younger than 70 years was identified and paired with a comparable group of patients, known as Group B, who were older than 70 years. Continuous monitoring of physiological parameters was conducted, and blood samples were collected at consistent intervals.

**Results:**

Totally, 188 participants were enrolled and received REBOA. Among the 188 patients, seventeen were aged more than 70 years. By implementing REBOA, the average amount of blood loss was only 1427 ml. Experiments were also conducted to compare Group A and Group B. No notable differences were observed in terms of demographic variables, systolic blood pressure (SBP), arterial pH, lactate levels, blood creatinine levels, potassium levels, or calcium levels at baseline. Additionally, after the deflation of the REBOA, laboratory test results, which included arterial pH, lactate, potassium concentration, calcium concentration, and blood creatinine concentration, were not significantly different (*P* > 0.05).

**Conclusion:**

This study indicated that in selected patients aged more than 70 years can achieve satisfactory hemodynamic and metabolic stability with Zone III REBOA.

**Level of evidence:**

Therapeutic study, Level III.

## Introduction

Lieutenant Colonel Hughes first described the use of the Resuscitative endovascular balloon occlusion of the aorta (REBOA) method during the Korean War [1]. This method entails the inflation of a balloon within a blood vessel to achieve control over bleeding. In recent decades, the REBOA technique has become popular in China and worldwide [2–6]. In the field of orthopedic tumors, resection of sacropelvic tumors is associated with a high risk of bleeding due to the rich blood supply in this anatomic location. To address this challenge, the technique of lower abdominal aortic balloon occlusion-Zone III REBOA- has been proven effective in managing hemorrhage during surgical procedures [2–4]. Zone III refers to the area positioned beneath the renal arteries yet proximal to the iliac bifurcation. This zone can be considered the least complex zone among the aorta and is designed for the purpose of controlling bleeding. Several clinical investigations [2, 3, 5, 7] have indicated that individuals who underwent REBOA experienced a notably shorter average duration of surgery, less blood loss, a diminished volume of blood transfusion, and a lower volume of postoperative drainage than individuals who did not undergo occlusion.

While REBOA is successful at achieving temporary control of bleeding, it carries risks related to certain factors and is not recommended in certain circumstances. Typically, it is recommended to refrain from using REBOA in patients who are older than 70 years or younger than 12 years [6]. Nonetheless, REBOA remains essential for reducing surgical blood loss in certain older patients with sacropelvic bone tumors. There is a lack of reported data on the clinical use of REBOA for elderly patients who underwent sacral and pelvic tumor removal. The aim of this research was to investigate the efficacy and safety of Zone III REBOA for performing sacral and pelvic tumor removal surgeries in patients aged > 70 years.

## Methods

### Study design and population

This study was conducted at a single center on musculuskeletal tumors in China. This study was approved by the Ethics Review Committee (ERC) of Peking University People’s Hospital (2020PHB107-01).

### Inclusion and exclusion criteria

The eligibility criteria for patients were as follows: (1) had tumors in the sacral or pelvic region; (2) were aged at least 18 years; (3) underwent complete tumor removal and reconstruction; (3) had the infrarenal aorta occluded once during the surgical procedure. The exclusion criteria were as follows: (1) patients who only received specialized medical treatment or biopsy and (2) patients with insufficient clinical data.

### Zone III REBOA technique

Previous reports have provided detailed information about Zone III REBOA [6, 8]. Briefly, the groin regions were sterilized after anesthesia induction. A percutaneous introducer sheath was placed in the femoral artery. Then, the balloon catheter was introduced through the arterial sheath. The position of the balloon catheter was confirmed by fluoroscopy. When the tumor was exposed but the hemorrhage was difficult to control, the balloon catheter was filled with normal saline to occlude the abdominal aorta.

The distal arterial pressure and urine volume were monitored constantly, which is obligatory and critical to guarantee that the balloon works properly. In the operating room, the nurse managed the device by inflating the balloon to the predetermined volume. Arterial blood gas readings, including glucose and electrolyte levels, were taken before surgery and after balloon deflation. The anesthesiologists monitored the intraoperative blood pressure, pulse rate, and hemoglobin levels. Every 30 min, 100 ml of saline containing 100 units of heparin was injected via the side arm of the femoral sheath catheter to prevent femoral artery thrombosis.

### Data points

We collected important data according to previous studies [3, 8, 9], including population characteristics (age, sex), tumor location, duration of surgery, duration of occlusion, and total volume of blood loss during surgery. Systolic blood pressure (SBP) was measured prior to inflating the balloon (baseline) and immediately after deflating the balloon following aortic occlusion. We assessed blood gas levels before and after occlusion. Baseline and post balloon deflation laboratory measurements, which included hemoglobin (Hb) levels, blood platelet, arterial pH, lactate levels, potassium (K^+^), calcium (Ca^2+^), serum creatinine (Scr), and prothrombin time (PT), were also obtained.

The amount of blood loss during surgery was calculated by summing the quantities of blood removed by mechanical suction and absorbed by dressings and sponges. Assessing the patient’s hemodynamic condition determined the necessity for a blood transfusion.

Balloon-related complication data, such as balloon migration/rupture, aortic rupture, puncture site hemorrhage, aortic aneurysms, acute femoral arterial thrombosis (distal embolus), extremity compartment syndrome, and acute kidney injury, were collected.

### Patient stratification

The patients were divided into two groups based on their age: individuals under 70 years old (Group A) and individuals over 70 years old (Group B).

### Statistical analysis

The statistical data were analyzed. The mean and standard deviation (SD) were used to report continuous parametric data. Categorical data are reported as a proportion. To compare the two groups, a χ2 test is used for categorical variables, a Mann‒Whitney test is used for continuous nonparametric data, and a t test is used for continuous parametric data. Statistical significance was determined for all *P* values from 2-sided tests with a threshold of *P* < 0.05. SPSS, Inc., was used to perform all the statistical analyses utilizing version 22 of the software.

## Results

In total, 188 participants were enrolled and received REBOA. Among the 188 patients, seventeen were aged more than 70 years. All patients underwent the surgeries, and thers was no perioperative death. Table 1 provides a summary of the demographic information. Among these patients, 56 were diagnosed with chondrosarcoma, 29 with metastatic lesions, 21 with giant cell tumors of the bone, 18 with osteosarcoma, 13 with neurofibroma/schwannoma, 9 with Ewing sarcoma, 5 with fibrosarcoma, and 37 with orthers. Group A consisted of 73 females and 98 males, while Group B consisted of 9 females and 8 males. There were no significant differences observed between Group A and Group B in terms of sex (98 men [57.3%] vs. 8 men [47.1%]; *P* = 0.416), BMI (24 [4] vs. 23 [3]; *P* = 0.375), mean operation time (239 [85] vs. 220 [97] min; *P* = 0.452), mean occlusion time (77 [27] vs. 65 [25] min; *P* = 0.091), mean preoperative SBP (108 [12] vs. 110 [16] mmHg; *P* = 0.732), average preoperative Hb (127 [19] vs. 128 [12] g/L; *P* = 0.665), average preoperative pH (7.4 [0.04] vs. 7.4[0.03]; *P* = 0.856), average preoperatvie lactate (0.9 [0.4] vs. 0.9 [0.4] mmol/L; *P* = 0.760), average preoperative potassium (4.0 [0.3] vs. 4.0 [0.4] mmol/L; *P* = 0.956), average preoperative calcium (2.3 [0.1] vs. 2.3 [0.1] mmol/L; *P* = 0.722), and average preoperative Scr (65 [15] vs. 72 [29] umol/L; *P* = 0.317).

**Table 1:** Characteristics of the two groups

Variables	Patients, No.		*P* Value
	Group A (*n* = 171)	Group B (*n* = 17)	
Age, mean (SD), y	44 (15)	73 (2)	< 0.05
Male sex (%)	98 (57.3)	8 (47.1)	0.416
BMI, mean (SD)	24 (4)	23 (3)	0.375
Operation time, mean (SD), min	239 (85)	220 (97)	0.452
Occlusion time, mean (SD), min	77 (27)	65 (25)	0.091
SBP at baseline, mean (SD), mmHg	108 (12)	110 (16)	0.732
Hb at baseline, mean (SD), g/L	127 (19)	128 (12)	0.665
Blood platelet at baseline, mean (SD), ×10^9^/L	248 (83)	209 (36)	0.062
Arterial pH at baseline, mean (SD)	7.4 (0.04)	7.4 (0.03)	0.856
Lactate at baseline, mean (SD), mmol/L	0.9 (0.4)	0.9 (0.4)	0.760
K^+^at baseline, mean (SD), mmol/L	4.0 (0.3)	4.0 (0.4)	0.956
Ca^2+^ at baseline, mean (SD), mmol/L	2.3 (0.1)	2.3 (0.1)	0.722
Scr at baseline, mean (SD), umol/L	65 (15)	72 (29)	0.317
PT at baseline, mean (SD), sec	11.8 (0.9)	11.8 (0.8)	0.857

The use of REBOA led to a notable increase in SBP from 108.26 mmHg (SD, 12.56) to 123.94 mmHg (SD, 16.16), which then decreased to 96.50 mmHg (SD, 9.55) mmHg immediately after deflation of the balloon (*P* < 0.05). The patient’s blood pressure remained consistent throughout the procedure. By implementing REBOA, the average amount of blood lost was merely 1427 ml (SD, 1008; range, 200–7900), while the average duration of the entire operation was 237 min (SD, 86; range, 85–600). Additionally, the mean duration of occlusion was 76 min (SD, 27; range, 10–150). Obviously, REBOA assisted the orthopedist in identifying clearly the surgical margin and neurovascular structure surrounded by the tumors. Furthermore, the occurrence of intraoperative contamination was reduced due to the decreased amount of intraoperative bleeding.

Upon deflation of REBOA, there was a significant decrease in Hb levels from 126.65 to 111.04 g/L (*P* < 0.05). The pH of the arteries was below the initial level (7.41 vs. 7.36, *P* < 0.05). The concentration of lactate in the arteries increased significantly from 0.92 to 1.47 (*P* < 0.05). The patient’s serum potassium levels rose from 3.95 to 4.12 mmol/L (*P* < 0.05). The serum calcium concentration decreased significantly from 2.29 to 2.02 mmol/L (*P* < 0.05). The level of creatinine in the blood decreased significantly from 65.57 to 63.35 µmol/L (*P* < 0.05) according to Table 2.

**Table 2:** Variables recorded just before balloon inflation (baseline) and just following balloon deflation in all patients

Variables	Baseline	Following balloon deflation	*P* value
SBP, mean (SD), mmHg	108.26 (12.56)	96.50 (9.55)	<0.05
Hb, mean (SD), g/L	126.65 (18.79)	111.04 (17.41)	<0.05
Arterial pH	7.41 (0.36)	7.36 (0.39)	<0.05
Lactate, mean (SD), mmol/L	0.92 (0.35)	1.47 (0.64)	<0.05
K^+^, mean (SD), mmol/L	3.95 (0.32)	4.12 (0.43)	<0.05
Ca^2+^, mean (SD), mmol/L	2.29 (0.13)	2.02 (0.14)	<0.05
Scr, mean (SD), umol/L	65.57 (17.05)	63.35 (18.33)	<0.05

### Complications associated with REBOA

Three patients experienced local hematoma at the puncture site. Lower limb ischemia (acute arterial thrombosis) occurred in three patients in Group A but not in Group B. By performing an embolectomy, the medical team successfully preserved the limb of the patient suffering from limb ischemia. No patients died during the perioperative period.

### Group A vs. Group B

Experiments were also conducted to compare Group A and Group B. No notable variations were observed in terms of demographic variables, SBP, arterial pH, lactate levels, blood creatinine levels, potassium levels, or calcium levels at the beginning of the study. Moreover, the laboratory tests conducted after the deflation of REBOA showed no significant differences in arterial pH, lactate, potassium, calcium, or blood creatinine levels (*P* > 0.05) (Table 3).

**Table 3:** Data from the two groups after deflation

Variables	Patients, No.		*P* Value
	Group A (*n* = 171)	Group B (*n* = 17)	
SBP after deflation, mean (SD), mmHg	96.57 (9.11)	95.76 (13.49)	0.740
Hb after deflation, mean (SD), g/L	111.26 (17.66)	108.76 (14.83)	0.574
Arterial pH after deflation	7.36(0.04)	7.36(0.04)	0.985
Lactate after deflation, mean (SD), mmol/L	1.48 (0.65)	1.34(0.56)	0.367
K^+^after deflation, mean (SD), mmol/L	4.16 (0.36)	4.27 (0.68)	0.353
Ca^2+^ after deflation, mean (SD), mmol/L	2.03 (0.14)	2.01 (0.13)	0.547
Scr after deflation, mean (SD), umol/L	62.61 (15.89)	70.76 (34.36)	0.347
Blood loss, mean (SD), ml	1412.05 (997.15)	1576.47 (1135.52)	0.523

## Discussion

The advantages of utilizing REBOA primarily stem from its minimally invasive nature and its ability to temporarily block the abdominal aorta. The applicability and potential of REBOA in adult trauma settings have garnered significant attention in recent years, although its effectiveness in elderly patients has largely been unexplored. A major obstacle to the widespread acceptance of REBOA is the insufficient availability of data. This study, which was conducted at a single center, examined the effects of Zone III REBOA on sacral and pelvic tumor resection in patients older than 70 years and revealed that the outcomes were satisfactory compared to those in patients younger than 70 years. To our knowledge, there have been no previous reports of these comprehensive findings on hemodynamics and metabolism.

By implementing REBOA in Zone III, the mean intraoperative blood loss was a mere 1427 ml, while the overall duration of the operation was 237 min. Although it is hard to standardize the surgeries of pelvic and sacral tumor, our results are significantly lower compared to an average blood loss of 4359 ml obtained in a group of 24 patients with conventional surgery without REBOA [10]. A previous study conducted in our center, a large series of 215 patients received sacral tumor resection surgery [4]. The reported mean blood loss in patients with REBOA was 2236 ml, whereas it was 3935 ml in patients without REBOA. There was a significant variability in the blood loss. It may be affected by many factors including the tumor location, the histology of the tumor, and the type of reconstruction. Similar to other investigations [4, 6, 11], the technique of Zone III REBOA can control bleeding during surgery that helps surgeons clearly identify the tumor mass and distinguish the surrounding neurovascular structure. Furthermore, the occurrence of intraoperative contamination was also reduced [2, 7, 11, 12]. The findings of this research support the existing guidelines for utilizing lower abdominal aortic balloon occlusion as an efficient method to substantially decrease blood loss while also providing surgeons with a clearer surgical field [2–5, 11].

According to certain researchers, it is advised to avoid exceeding a duration of 60 min for aortic occlusion in Zone III of the aorta [13]. Nevertheless, our previous research demonstrated that a 90-minute occlusion of REBOA resulted in satisfactory hemodynamic and metabolic stability, despite prolonged occlusion leading to comparatively elevated levels of lactate [8]. Typically, REBOA is employed for highly vascular tumors (such as giant cell tumors or metastatic renal cancers), initial malignant tumors, and recurring tumors. However, REBOA was not used in patients who had aortic dissection or aneurysm (which was strictly contraindicated), renal artery bifurcation below the L2 to L3 disc, a history of previous surgeries involving balloon use, or unstable plaque detected on abdominal CT. Usually, a history of hypertension was not a absolute contraindication for the use of REBOA, which can be managed by anesthetists during surgery. Patients older than 70 years are generally in poor health and have poor tolerance to surgery. Nevertheless, during this investigation, elderly individuals underwent procedures without any major complications.

Vascular complications associated with balloons include hematoma occurring at the puncture location and acute formation of arterial blood clots. Interestingly, three acute arterial thromboses occurred in Group A but not in Group B; this could be explained by the small sample size in Group B. Surgeons performing REBOA should be mindful of these potential access site consequences, which are typically a result of the puncture technique rather than being specific to REBOA. It is crucial for these patients to address these complications during sheath removal to prevent limb-threatening vascular complications [14]. The Seldinger technique allows for the placement of an REBOA catheter by puncturing the femoral artery with a hollow needle, passing a guidewire through the needle, and subsequently exchanging the needle for an introducer sheath [15]. The procedure can be carried out by using anatomical reference points and manually detecting the common femoral artery, or it can be performed with the assistance of ultrasound. Surgical cut-down can also achieve cannulation if necessary [16]. Ultrasound, plain X-ray, or fluoroscopy can be used to confirm the location of the guidewire.

The understanding of the impact of aortic occlusion on overall blood clotting and inflammation is limited due to the challenges in comprehending the precise effects of REBOA in the context of bleeding and the restoration of blood flow after ischemia. Determining the maximum ischemic threshold during REBOA becomes challenging due to these perplexing factors. In this study, laboratory measurements, such as arterial pH, potassium, calcium, and serum creatinine, were similar for both groups of patients (*P* > 0.05).

Deflation of the balloon results in ischemia and reperfusion, which triggers reactive hyperemia through the inflammatory response; this leads to blood pooling in the distal regions of the body, causing a significant decrease in SBP [17]. Nevertheless, the temporary low blood pressure following deflation was elevated to the initial level subsequent to the administration of a rapid infusion of crystalloid solution and transfusion of blood. In elderly patients, blood pressure remains consistent throughout surgery.

Some limitations exist in this study we must ackowlege. First, it is a retrospective study with a small number of cases. The study totally enrolled 188 patients and only 17 patients in Group B. Second, the heterogeneity of two groups do exist. The focus of this study was detection the feasibility and safety of Zone III REBOA in population that older than 70 years. Therefore, we only campare the parameter of age. Third, selection bia is inevitable in this study. If an elderly individual encounters a scenario where REBOA cannot be applied, then naturally REBOA may be not utilized. Actually, the strictly contraindication of REBOA were aortic dissection or aneurysm. Most of ptients with sacropelvic bone tumors will received REBOA. Fourth, this study lack comparison group with patients treated without REBOA.

Group A and Group B did not show any notable variations in terms of demographics, SBP, arterial pH, lactate, blood creatinine, potassium, or calcium at the beginning of the study. Moreover, while decompressing the REBOA, the laboratory assessments, encompassing arterial pH, lactate, potassium, calcium, and blood creatinine, exhibited identical values. The occlusion balloon of the lower abdominal aorta was situated distal to the superior mesenteric artery and the renal artery. As a result, blood flow to organs in the abdomen and the kidney is not impaired. Hence, employing Zone III REBOA in selected individuals aged > 70 years who underwent sacral and pelvic tumor removal surgeries proves to be a safe and efficient technique for minimizing hemorrhage.

## Data Availability

The datasets used and/or analyzed during the current study are available from the corresponding author on reasonable request.
